# Relationship of vitamin D receptor gene polymorphism with sarcopenia and muscle traits based on propensity score matching

**DOI:** 10.1002/jcla.23485

**Published:** 2020-07-22

**Authors:** Xuemei Yao, Lei Yang, Meiyan Li, Hui Xiao

**Affiliations:** ^1^ Department of Epidemiology and Biostatistics School of Public Health Xinjiang Medical University Urumqi China

**Keywords:** muscle traits, sarcopenia, VDR gene polymorphism

## Abstract

**Background:**

Vitamin D receptor (VDR) gene polymorphism is reported to be associated with muscle mass and muscle strength. Loss of skeletal muscle mass and decreased muscle strength are the main characteristics of sarcopenia. In this study, the relationship of VDR gene polymorphism with muscle traits (muscle mass, muscle strength, and physical performance) and sarcopenia were studied in Xinjiang, China.

**Methods:**

Totally, 205 sarcopenia patients were enrolled. Propensity score method was used to match control group. FokI and BsmI polymorphisms were genotyped using improved multiplex ligation detection reaction (iMLDR).

**Results:**

Fok1, but not Bsm1, polymorphism was significantly associated with sarcopenia. Patients with Fok1 GG genotype were more likely to have sarcopenia. Both Bsm1 and Fok1 polymorphism were related to muscle traits. Patients with Bsm1 CT genotype had lower gait speed (GS) but higher skeletal mass index. Patients with Fok1 GG genotype had lower GS, and female subjects with the Fok1 GG genotype had lower handgrip strength (HS). GS was decreased in Bsm1 CT genotype than CC carriers. HS and GS were decreased in Fok1 GG genotype than AA carriers.

**Conclusion:**

Fok1, but not Bsm1, polymorphism is associated with sarcopenia. Both Bsm1 and Fok1 polymorphism affect both HS and GS.

## Graphical abstract

Fok1 polymorphism affects both handgrip strength and gait speed. Bsm1 polymorphism affects gait speed. But only Fok1 is associated with sarcopenia.

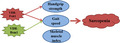

## 1 INTRODUCTION

Sarcopenia is a degenerative disease that causes loss of skeletal muscle mass and decreased muscle function (eg, decreased muscle strength and gait speed (GS)).^1^ Epidemiological data show that sarcopenia is much more common in the older adults.^2^ A 4‐year longitudinal study showed that compared with black people and Caucasian, the older Chinese had less muscle mass, weaker grip strength, and slower GS.^3^ The prevalence of sarcopenia in China ranges from 9% to 28%.^4^, ^5^, ^6^ People with sarcopenia are more likely to become frailty and disabled, as well as have declined life quality.^7^, ^8^, ^9^, ^10^, ^11^ Therefore, sarcopenia has attracted attention from researchers all over the world.

The pathophysiology of sarcopenia involves many factors. For example, prevalence of sarcopenia varies in population with different demographic characteristics (such as age and sex).^6^ Sarcopenia is also related to nutrition status (eg, vitamin D level),^6^, ^12^, ^13^ physical activity,^13^, ^14^, ^15^ and sleep quality.^4^, ^16^, ^17^ On the other hand, a number of studies have shown that skeletal muscle strength and mass are highly heritable, indicating a strong genetic contribution.^18^, ^19^ However, only a few genes have been confirmed to be related to skeletal muscle strength and mass. There are few reports on the association of genetic polymorphisms with sarcopenia.

There is a certain relationship among skeletal muscle mass, strength, and vitamin D levels, as well as vitamin D receptor (VDR) gene polymorphisms. Studies have shown that low vitamin D is associated with low muscle strength, muscle mass,^20^ and muscle function.^21^ Geusens et al ^22^ found that grip strength was associated with VDR genotype in older women (age ≥ 70 years). The Fok1 and Bsm1 loci of the VDR gene are associated with muscle mass and muscle strength. Subjects with the FF/AA genotype of Fok1 were more likely to have higher skeletal muscle strength and quality than FF/GG carriers.^23^, ^24^, ^25^ Meanwhile, the carriers with BB/CC genotype of Bsm1 were more likely to have higher skeletal muscle strength than those with BB/TT genotype.^25^, ^26^ All these studies have highlighted the role of VDR gene polymorphisms in muscle mass and muscle strength.

Randomized controlled trial (RCT) is considered as the optimal study method.^27^ However, RCT is subject to ethical, economic, and other conditions. Propensity score matching (PSM) aims to reduce or eliminate differences between baseline features.^28^ PSM method can effectively balance the confounding factors between the diseased group and the control group. Compared to RCT, PSM is widely used in non‐RCT.

Here, in this study, we studied the relationship of VDR gene polymorphism with muscle traits (muscle mass, muscle strength, and physical performance) and sarcopenia. The subjects were Urumqi residents from Tianshan district of Xinjiang, China. The control group was matched by PSM method (in 1:2 matching).

## 2 MATERIALS AND METHODS

### 2.1 Ethics

All subjects provided written informed consent. The study was conducted in accordance with the Declaration of Helsinki, and the study protocol was approved by the Ethics Committee of Xinjiang Medical University.

### 2.2 Subjects

This study was carried out between March 2017 and July 2018. A total of 1886 subjects were enrolled in this study. They were subjects who underwent physical examination in Tianshan district of Urumqi, China.

The inclusion criteria were as follows: (a) Urumqi residents, (b) aged ≥50 years. The exclusion criteria were as follows: (a) subjects with limited mobility, (b) subjects with disorder of communication or mental illness, (c) subjects with hyperthyroidism or hypothyroidism, (d) subjects with long‐term use of steroids, (e) subjects taking weight‐loss drugs and glucocorticoids that might affect body composition in the past three weeks, (f) subjects with metal stents or pacemaker, and (g) physically disabled or amputee.

Asian Working Group for Sarcopenia (AWGS) ^29^ defined sarcopenia as reduction in skeletal muscle mass, skeletal muscle strength, and/or physical performance. According to AWGS, 205 subjects were diagnosed as sarcopenia in our study and 410 subjects were matched by PSM method (in 1:2 matching) as the control group. The matched covariate variables included age, sex, ethnicity, educational level, monthly income, marital status, smoking, and alcohol consumption.

### 2.3 Determination of Sarcopenia

#### 2.3.1 Body composition

The appendicular skeletal muscle mass (ASM) was measured using bioelectrical impedance analysis on a body composition analyzer (InBody 720, Korea). During measurement, the participants stood on the device in bare feet with legs slightly apart while holding the electrodes in both hands with arms away from the trunk. The skeletal mass index (SMI) was defined as ASM/height.^2^ We used the AWGS to determine the cutoff value of SMI, which is 7.0 and 5.7 kg/m^2^ for male subjects and female subjects, respectively.

#### 2.3.2 Muscle strength (Hand grip strength)

Muscle strength was assessed by HS. HS was tested three times for the dominant and non‐dominant hand using Jamar Hand Dynamometer (Sammons Preston Inc) with a break of 30 seconds between each test. Grip width was adjusted individually. The average value of the three tests was used for analysis. According to AWGS, the cutoff value of HS is 26 kg and 18 kg for male subjects and female subjects, respectively.

#### 2.3.3 Physical performance (gait speed)

Physical performance was examined by 10‐meter GS.^29^ In order to allow participants to accelerate/decelerate outside the data collection area, 5‐meter distance was provided at the beginning and end of the walkway, respectively. We marked 4 positions (0, 5, 15, and 20 m), and walking time was collected from 5 to 15 m. Participants were instructed to “walk at your usual pace.” Then, GS was calculated as 10(m)/time(s). According to AWGS, the cutoff value of GS is 0.8m/s for both male subjects and female subjects.

Sarcopenia was defined as presentation of both low muscle mass and function (strength or performance) following the AWGS criteria.

### 2.4 Genotyping

Blood samples were collected during physical examination. Genomic DNA was extracted using the Whole Blood Extraction kit (Tiangen Biotech). The Fok1 and Bsm1 genotyping were performed using improved multiplex ligation detection reaction (iMLDR, Genesky, Shanghai, China). Briefly, the genomic DNA was amplified using the PCR instrument (Mycycler, Bio‐Rad). The procedure was 25 cycles of 96°C for 10 seconds, 50°C for 5 seconds, and 60°C for 30 seconds. PCR product (10 μL) was then digested with enzymes. The digested fragments were subjected to electrophoresis on 2% agarose gels and visualized with UV light. A double‐blind control was set up during the genetic polymorphism detection, and 1% of the samples were randomly selected for repeated detection.

### 2.5 Statistical analyses

R statistical software was used to match case and control group (in 1:2 matching). Caliper matching method was used, and the caliper value was 0.2. All other data were analyzed by SPSS21.0 (IBM). Shapiro‐Wilk method was used to test normality of quantitative data. If normality was satisfied, the data were described as mean ± standard deviation (SD). Qualitative data were described as percentage. Chi‐square (χ^2^) test was used to analyze categorical variables (ie, genotype and Hardy‐Weinberg equilibrium). Univariate logistic regression was used to determine the effect of different genotypes on the risk of sarcopenia. The odds ratios (OR) and 95% confidence intervals (CI) were calculated. The comparisons of HS, GS, and SMI between different genotypes were conducted with one‐way analysis of variance (ANOVA) followed by the student‐newman‐keuls method. Multivariate linear regression was used to analyze the association of different genotypes with HS, GS, and SMI. In Model 1, the confounding factor was not adjusted; in Model 2, the confounding factors were adjusted, including age, sex, ethnicity, educational level, monthly income, marital status, smoking, and alcohol consumption. A *P*‐value <.05 was considered statistically significant.

## 3 RESULTS

### 3.1 Comparisons of covariate before and after PSM

**Figure 1:**
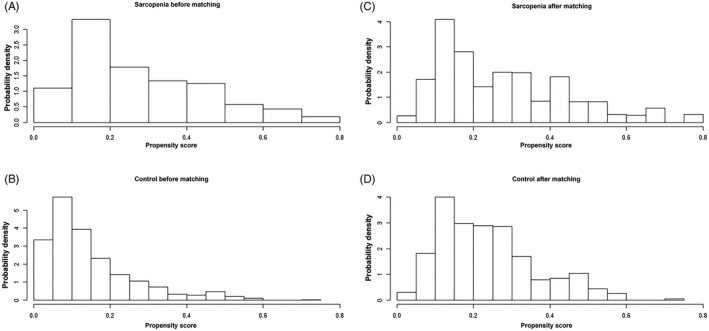
Histogram of propensity score before and after matching. This figure mainly focuses on the distribution of PS scores before and after matching case group and the control group. A, Sarcopenia group before matching. B, Control group before matching. C, Sarcopenia group after matching. D, Control group after matching

**Figure 2:**
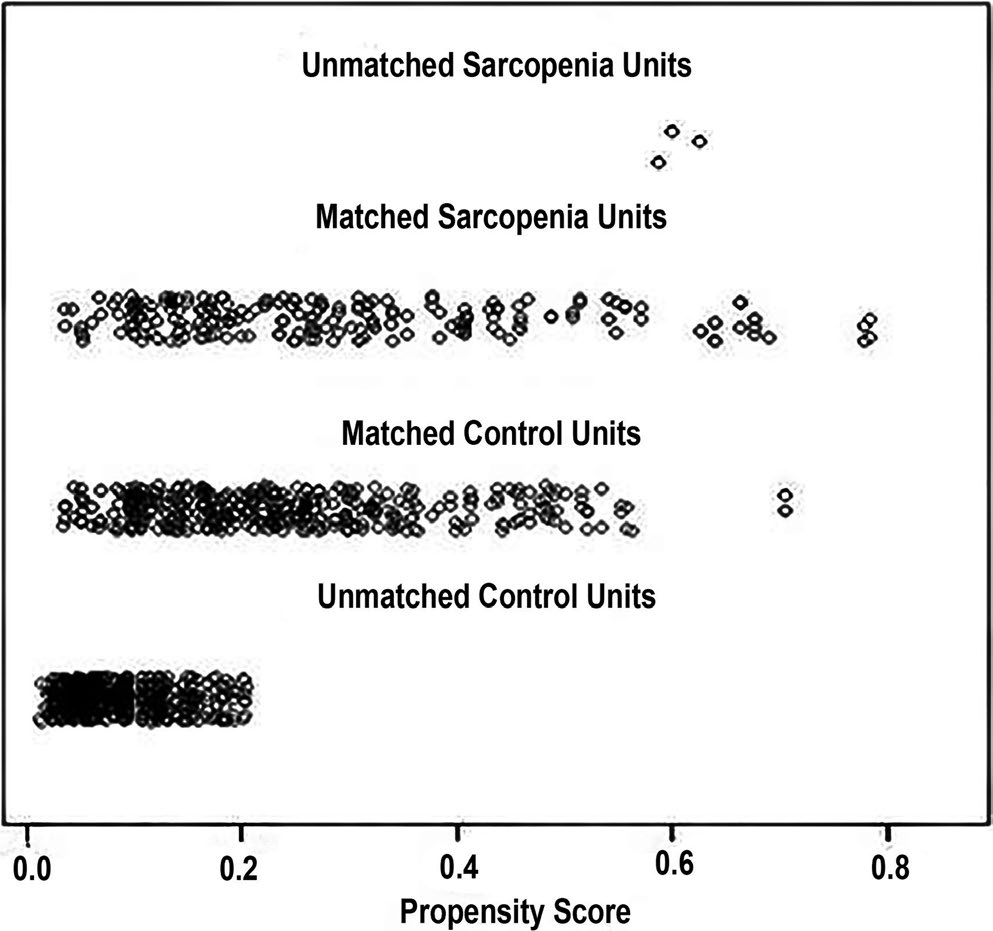
Jetter plot of propensity score matching. This figure mainly focuses on whether the matching of the case control is complete and the distribution of PS scores in the two groups after the successful matching

As shown in Table 1, there was a significant difference in age, ethnicity, educational level, marital status, and alcohol consumption between sarcopenia and control groups (*P* < .05) before PSM. However, there was no significant difference in them between the two groups after PSM. The distribution of PS consistently demonstrated an improvement in the covariate balance after PSM (Figures 1 and 2).

**Table 1:** Comparison of covariate before and after PSM

Covariates	Before PSM			After PSM		
	Control (n = 1678)	Sarcopenia (n = 208)	*P*	Control (n = 410)	Sarcopenia (n = 205)	*P*
Age (years)	62.98 ± 7.30	68.97 ± 7.01	**<.001**	67.92 ± 7.43	68.77 ± 6.86	.170
Sex						
Male	687 (87.6)	97 (12.4)	.116	183 (44.6)	94 (45.9)	.774
Female	991 (89.9)	111 (10.1)		227 (55.4)	111 (54.1)	
Ethnicity						
Han	1431 (89.0)	177 (11.0)	.944	345 (84.1)	174 (84.9)	.814
Minority	247 (88.8)	31 (11.2)		65 (15.9)	31 (15.1)	
Educational level						
High school and below	964 (57.4)	145 (69.7)	**.002**	267 (65.1)	142 (69.3)	.488
Technical college or equivalent	569 (33.9)	53 (25.5)		115 (28.0)	53 (25.9)	
Undergraduate and above	145 (8.6)	10 (4.8)		28 (6.8)	10 (4.9)	
Monthly income (RMB)						
<3000	138 (89.0)	17 (11.0)	**.006**	28 (6.8)	17 (8.3)	.540
3000～3999	435 (87.0)	65 (13.0)		142 (34.6)	64 (31.2)	
4000～4999	710 (87.8)	99 (12.2)		199 (48.5)	97 (47.3)	
≥5000	395 (93.6)	27 (6.4)		41 (10.0)	27 (13.2)	
Marital status						
Married	1461 (91.0)	145 (9.0)	**<.001**	315 (76.8)	145 (70.7)	.101
Divorced/widowed	217 (77.5)	63 (22.5)		95 (23.2)	60 (29.3)	
Smoking						
No	1245 (88.9)	155 (11.1)	.920	322 (78.5)	88 (21.5)	.340
Yes	433 (89.1)	53 (10.9)		154 ( (75.1)	51 (24.9)	
Alcohol consumption						
No	1547 (88.3)	204 (11.7)	**.002**	403 (98.3)	201 (98.0)	.830
Yes	131 (97.0)	4 (3.0)		7 (1.7)	4 (2.0)	

Abbreviation: PSM, Propensity score matching.

The bold values present these comparisons were statistically significant.

### 3.2 Hardy‐Weinberg equilibrium tests and the distribution of the Bsm1 and Fok1 genotypes

The distribution of the Bsm1 and Fok1 genotypes met with Hardy‐Weinberg equilibrium (*P* > .05). As shown in Table 2, the frequency of Bsm1 genotypes CC, CT, and TT was 83.4%, 16.6%, and 0.0% in control group and 80.5%, 18.5%, and 1.0% in sarcopenia group. The frequency of Fok1 genotypes AA, GA, and GG was 25.1%, 49.0%, and 25.9% in control group and 21.5%, 42.9%, and 35.6% in sarcopenia group. The distribution of Fok1 genotypes was significantly different between sarcopenia and control groups (*P* < .05). Similarly, the results of logistic regression analysis showed that GG genotype increased the risk of sarcopenia compared to AA genotype (OR = 1.612, 95% CI: 1.016, 2.559) and that G‐allele increased the risk of sarcopenia compared to A allele (OR = 1.310, 95% CI: 1.032, 1.663). However, the Bsm1 gene polymorphism was not significantly associated with sarcopenia (Table 2).

**Table 2:** Genotype and allele distribution of VDR in sarcopenia and control groups

SNP	Genotype/Allele	Control	Sarcopenia	χ^2^	*P*	*OR* (95%*CI*)
Bsm1	CC	342 (83.4)	165 (80.5)	4.444	.108	1
	CT	68 (16.6)	38 (18.5)			1.158 (0.747, 1.795)
	TT	0 (0.0)	2 (1.0)			–
	C	752 (91.7)	368 (89.8)	1.278	.258	1
	T	68 (8.3)	42 (10.2)			1.262 (0.842, 1.891)
χ^2^ and *P*‐value for HWE		3.352/0.067				
Fok1	AA	103 (25.1)	44 (21.5)	6.316	**.043**	1
	GA	201 (49.0)	88 (42.9)			1.025 (0.665, 1.580)
	GG	106 (25.9)	73 (35.6)			**1.612 (1.016, 2.559)**
	A	407 (49.6)	176 (42.9)	4.932	**.026**	1
	G	413 (50.4)	234 (57.1)			**1.310 (1.032, 1.663)**
χ^2^ and *P*‐value for HWE		0.013/0.909				

Abbreviations: HWE, Hardy‐Weinberg equilibrium; VDR, vitamin D receptor.

The bold values present these comparisons were statistically significant.

### 3.3 Comparison of HS, GS, and SMI across the Bsm1 and Fok1 genotypes

Comparisons of HS, GS, and SMI among the three Bsm1 genotypes by sex were showed in Table 3. The results showed that there was lower GS in Bsm1 CT carriers compared to CC carriers (0.91 ± 0.17 vs 1.01 ± 0.21, *P* = .006) in male subjects. However, higher SMI was found in Bsm1 CT carriers compared to CC carriers (7.77 ± 1.12 vs 7.37 ± 1.03, *P* = .036). HS, GS, and SMI of different Bsm1 genotypes had no significant differences in female subjects (*P* > .05).

**Table 3:** Comparison of HS, GS, and SMI for genotypes of Bsm1

Sex		CC	CT	TT	*F*	*P*
Male	HS (kg)	32.26 ± 7.73	30.84 ± 6.36	–	1.113	.293
	GS (m/s)	1.01 ± 0.21	0.91 ± 0.17	–	7.751	**.006**
	SMI (kg/m^2^)	7.37 ± 1.03	7.77 ± 1.12	–	4.475	**.036**
Female	HS (kg)	19.82 ± 4.82	19.79 ± 4.65	19.76 ± 3.96	1.326	.267
	GS (m/s)	0.96 ± 0.22	0.94 ± 0.23	0.93 ± 0.10	0.205	.815
	SMI (kg/m^2^)	5.98 ± 0.70	6.08 ± 0.78	6.64 ± 0.53	0.795	.452

Abbreviations: GS, gait speed; HS, handgrip strength; SMI, skeletal mass index.

The bold values present these comparisons were statistically significant.

Comparisons of HS, GS, and SMI among the three Fok1 genotypes by sex were showed in Table 4. The GS for male subjects and the HS for female subjects were significantly different among Fok1 genotypes (*P* < .05). Multiple comparisons revealed that there was lower GS in Fok1 GG carriers compared to AA and GA carriers (GG: 0.93 ± 0.17, AA: 0.99 ± 0.17, GA: 1.03 ± 0.23, *P* = .013) in male subjects. And for female subjects, lower HS in Fok1 GG carriers was observed compared to AA and GA carriers (GG: 18.68 ± 4.52, AA: 20.31 ± 4.80, GA: 20.10 ± 4.83, *P* = .047).

**Table 4:** Comparison of HS, GS, and SMI for genotypes of Fok1

Sex		AA	GA	GG	*F*	*P*
Male	HS (kg)	32.34 ± 8.18	32.65 ± 7.74	30.68 ± 6.28	1.313	.271
	GS (m/s)	0.99 ± 0.17	1.03 ± 0.23	0.93 ± 0.17	4.410	**.013**
	SMI (kg/m^2^)	7.53 ± 1.16	7.51 ± 1.03	7.26 ± 0.97	1.245	.290
Female	HS (kg)	20.31 ± 4.80	20.10 ± 4.83	18.68 ± 4.52	3.078	**.047**
	GS (m/s)	0.99 ± 0.21	0.96 ± 0.23	0.93 ± 0.20	1.897	.152
	SMI (kg/m^2^)	5.91 ± 0.71	6.07 ± 0.71	5.94 ± 0.72	1.641	.196

Abbreviations: GS, gait speed; HS, handgrip strength; SMI, skeletal mass index.

The bold values present these comparisons were statistically significant.

### 3.4 Multivariate analyses of relationship of the Bsm1 and Fok1 genotypes with HS, GS, and SMI

Multivariate linear regression analyses of the association between Bsm1 genotypes and HS, GS, and SMI were shown in Table 5. The results showed that subjects with Bsm1 CT genotype were more likely to have less GS compared to those with CC genotype (β = −0.05, 95% CI: −0.10, −0.01). There was still statistical significance after adjusting for confounding factors, including age, sex, ethnicity, educational level, monthly income, marital status, smoking, and alcohol consumption (β = −0.06, 95%CI:‐0.11, −0.02).

**Table 5:** The correlation of HS, GS, and SMI to Bsm1 genotypes

	Bsm1		
	CC	CT	TT
HS			
Model 1	1	−0.16 (−2.13, 1.80)	−12.72 (−29.45,4.02)
Model 2	1	−0.78 (−2.17, 0.61)	−6.88 (−18.46, 4.70)
GS			
Model 1	1	−**0.05 (−0.10, −0.01)**	−0.05 (−0.47, 0.37)
Model 2	1	−**0.06 (−0.11, −0.02)**	−0.01 (−0.41, 0.39)
SMI			
Model 1	1	**0.28 (0.03, 0.54)**	0.11 (−2.09 2.31)
Model 2	1	0.12 (−0.08, 0.32)	0.32 (−1.36, 2.00)

Note

Model 1: Confounding factor was not adjusted. Model 2: Confounding factors were adjusted, including age, sex, ethnicity, educational level, monthly income, marital status, smoking, and alcohol consumption.

The bold values present these comparisons were statistically significant.

Multivariate linear regression analyses of the association between Fok1 genotypes and HS, GS, and SMI were shown in Table 6. In Model 1, subjects with Fok1 GG genotype were more likely to report less GS compared to those with AA genotype (β = −0.06, 95%CI: −0.11, −0.01). In Model 2, after adjusting for confounding factors, subjects with GG genotype had lower HS (β = −1.58, 95%CI:‐2.97, −0.18) and GS (β = −0.05, 95%CI: −0.10, −0.01) compared to those with AA genotype.

**Table 6:** The correlation of HS, GS, and SMI to Fok1 genotypes

	Fok1		
	AA	GA	GG
HS			
Model 1	1	−1.52 (−3.35, 0.31)	−2.64 (−4.66, −0.62)
Model 2	1	−0.35 (−1.62, 0.93)	−**1.58 (−2.97, −0.18)**
GS			
Model 1	1	−0.00 (−0.05, 0.05)	−**0.06 (−0.11, −0.01)**
Model 2	1	−0.01 (−0.06, 0.03)	−**0.05 (−0.10, −0.01)**
SMI			
Model 1	1	−0.10 (−0.34, 0.14)	−0.22 (−0.49, 0.04)
Model 2	1	0.05 (−0.14, 0.23)	−0.10 (−0.30, 0.10)

Note

Model 1: Confounding factor was not adjusted. Model 2: Confounding factors were adjusted, including age, sex, ethnicity, educational level, monthly income, marital status, smoking, and alcohol consumption.

The bold values present these comparisons were statistically significant.

## 4 DISCUSSION

The pathogenesis of sarcopenia is not clear. The association between VDR gene polymorphism and sarcopenia is less reported. In this study, we explored the association between the gene polymorphism of Bsm1 and Fok1 of VDR and sarcopenia, as well as muscle trait. We used the PSM method to match the sarcopenia group and control group. Prior to PSM, some demographic characteristics were unbalanced, which may affect the distribution of genes. After PSM, both groups showed similar demographic characteristics with no significant differences, indicating that PSM effectively minimizes the imbalance between covariates.

The level of VDR in the skeletal muscle of the older adults decreases with age and is lower than that of young people.^30^, ^31^ Previous epidemiological studies of the relationship between vitamin D levels and muscle characteristics have shown that low levels of vitamin D status are associated with lower muscle strength,^32^, ^33^, ^34^, ^35^ but not related to muscle mass.^35^ The effect of VDR gene polymorphism on muscle characteristics has been reported. In 1997, Geusens et al ^22^ reported for the first time that carriers of Bsm1 bb/CC genotype had higher quadriceps strength and grip strength than those of the BB/TT genotype. Xia et al ^25^ found that the Fok1 f/A allele and the Bsm1 bb/CC genotype carriers had higher grip strength. Our study showed that compared with CC genotype, GS and SMI were lower in Bsm1 CT genotype only for male subjects. This effect was only observed in GS after adjusting for confounding factors. Compared with AA genotype, GS was lower in Fok1 GG genotype for male subjects, and HS was lower for female subjects. After adjusting for confounding factors, both HS and GS were lower in subjects with Fok1 GG genotype. Our findings suggest that both Bsm1 and Fok1 of VDR are only related to muscle function of GS or HS, but not muscle mass of SMI. These results were similar to that of Meng et al,^35^ which also reported that serum 25(OH)D was positively associated with muscle function, but not muscle mass.

For patients with sarcopenia, their skeletal muscle mass and strength decrease with age. For patients with osteoporosis, their bones show similar age‐dependent changes. There are similar changes in aging population in muscles and bones.^36^ The association between VDR gene polymorphism and osteoporosis has been confirmed.^37^ Little research has been done on VDR gene polymorphisms and sarcopenia. Walsh et al ^24^ reported that the Fok1 FF/GG genotype was associated with an increased risk of sarcopenia in older women (1.3 times increased risk) compared with the f allele carrier, whereas Bsm1 was not associated with sarcopenia. In accordance with this research, our study observed that Fok1 GG genotype was more likely to increase the risk of sarcopenia when compared to AA genotype. Although the associations of Bsm1 with sarcopenia showed no statistical significance, CT genotype carriers exhibited significantly higher GS compared to CC genotype carriers.

However, our research has some limitations. First, in our study, there were no male cases in the comparison of HS, GS, and SMI in different genotypes of Bsm1. The results may be biased. Second, the participants in our study were recruited from physical examination centers. Therefore, there were sample bias in this study, including sample selection bias (eg, people with poor economic condition or limited mobility may be less likely to do health examination). Finally, the sample size was relatively small. Further studies with larger sample size are needed to verify the relationship of VDR gene polymorphisms with sarcopenia.

## 5 CONCLUSIONS

In conclusion, subjects with the Fok1 GG genotype are more likely to have sarcopenia. Even after adjusting potentially confounding factors, there is lower GS in Bsm1 CT compared to CC carriers and lower HS and GS in Fok1 GG compared to AA carriers. Our findings indicate that VDR genotypes can affect muscle traits and sarcopenia.

## AUTHOR CONTRIBUTIONS

HX involved in conceptualization, writing‐review and editing, supervision, project administration, and funding acquisition; XY, LY, and ML involved in methodology, software, and formal analysis; XY involved in data curation and writing‐original draft preparation.
